# Implementation of Goal-Oriented Care in Belgium: Experiences From 25 Primary Care Organisations

**DOI:** 10.5334/ijic.8983

**Published:** 2025-05-02

**Authors:** Lotte Vanneste, Isabelle Heymans, Jean-Luc Belche, Ine Huybrechts, Dominique Van de Velde, Patricia De Vriendt, Reini Haverals, Dagje Boeykens, Sibyl Anthierens, Pauline Boeckxstaens

**Affiliations:** 1 Department of Public Health and Primary Care, Faculty of Medicine and Health Sciences, Ghent University, Ghent, Belgium; 2 Department of Family Medicine, Faculty of Medicine, University of Liège, Liège, Belgium; 3 Department of Family Medicine and Population Health, University of Antwerp, Antwerp, Belgium; 4 Department of Family Medicine and Chronic Care, Faculty of Medicine and Pharmacy, Vrije Universiteit Brussel, Brussels, Belgium; 5 Department of Rehabilitation Sciences, Occupational Therapy, Faculty of Medicine and Health Sciences, Ghent University, Ghent, Belgium; 6 Expertise Centre Health and Care, Artevelde University of Applied Sciences, Ghent, Belgium; 7 Frailty in Ageing (FRIA) Research Group, Mental health and wellbeing (MENT) research group, Department of Gerontology, Faculty of Medicine and Pharmacy, Vrije Universiteit Brussel, Brussels, Belgium

**Keywords:** goal-oriented care, implementation, primary care, integrated care

## Abstract

**Introduction:**

Goal oriented care (GOC) and its readiness for implementation has been described in scientific literature, but research on GOC implementation in primary care organisations is limited. This study aims to capture the experiences of primary care organisations in implementing GOC in their context.

**Method:**

A qualitative study, with data triangulation, was conducted. Primary care organisations that experimented with the implementation of GOC in their context were followed. Data were analysed using inductive thematic analysis.

**Results:**

Seven themes supporting GOC implementation were identified. Project leaders from the primary care organisations experienced that related concepts can serve as a foundation for initiating the implementation of GOC. The implementation process is an iterative and reflective process, with resistance viewed as an integral part of the process, offering opportunities for reflection. Collaborating with partners, especially the active participation of patients, was seen as a facilitator. Furthermore, having a clear vision for GOC is necessary. Projects invested in adapting tools and processes to align with GOC and provided relevant training.

**Discussion and conclusion:**

The findings led to recommendations that can guide the implementation of future GOC projects. Effective implementation extends beyond the development and adaptation of tools; it requires translating theoretical concepts into practical application and creating a shared vision on GOC.

## Introduction

Healthcare systems are traditionally built and organised within a disease- and problem-oriented paradigm, which is increasingly being challenged by the complexity of the problems people face [1,2]. Goal-oriented care (GOC) is a concept of care in which the patient’s personal goals are put first in clinical decision making and in the organisation of care processes. In GOC, care is organised based on what is important to the patient and the patient’s values [3]. This model of care is presented as an alternative to the dominating problem-oriented and disease oriented care model and of particular value for the increasing numbers of people with complex health and social care needs [2,4]. GOC values embracing ethical principles such as autonomy and beneficence [2].

In the management of complex health and social care needs, the alignment of care is perceived as crucial [5,6]. Each disease is guided by its own specific set of guidelines and objectives, creating complexities that can hinder effective integration of care. Because of this complexity, it is important to align care to ensure continuity and prevent gaps [7]. Because personal goals start from the experience of patients, and are not disease based, this could help align the process of care. The personal goal is a common goal that is pursued by all persons and parties involved, regardless of disease goals. Therefore, it is suggested that GOC could be a driver for integrated care [8]. Emphasizing the patient’s personal goals, GOC drives integration at clinical, professional, organisational and system levels, encouraging a shared philosophy of care across different healthcare settings. The Rainbow Model for Integrated Care (RMIC) was used to support this rationale [8]. Key to the RIMC is that successful implementation of integrated care asks for interventions at different levels [9]. The micro level, or clinical integration level, refers to the process of individual care between patient and provider. The meso level refers to professional integration and organisational integration. At this level, professional integration relates to the collaboration between professionals and interprofessional partnerships. Organisational integration focusses on collaboration between organisations. The macro level refers to the governmental level and focuses on policy and systems integration. Underlying all these dimensions are normative integration and functional integration. Normative integration includes the shared concept and frame of reference, functional integration focuses on support functions to facilitate the process of care [8,9].

The concept of GOC and its readiness for implementation has been described by Huybrechts et al. who reported that primary care professionals recognise the value of GOC and are positively engaged in its practical implementation [10,11]. Primary care providers acknowledge the importance of the patient’s personal goals, they take time to put the goals first and use these to guide interprofessional collaboration [12]. Patients experience that professionals are putting the patient’s personal goals first, but they don’t experience if and how these goals are translated to care goals and a care plan and if these goals are evaluated [13]. In Belgium, the importance of policy support in implementing GOC is stated [11]. In 2023, a protocol for a Belgian integrated care plan was agreed upon by the federal government. In this plan, GOC is formulated as a lever for integrated care [14].

Despite this engagement towards GOC, primary care professionals experience challenges on the organisational level to adopt GOC in practice [12]. Current research on GOC is mostly focused on conveying the theoretical concept into real-world cases, in particular in terms of goal-setting and the translation of patient goals into care plans [15,16]. To our knowledge, there is limited research that describes the implementation of GOC within primary care organisations. The Dr Daniël De Coninck Fund, which is part of the King Baudouin Foundation a Belgian philantrophy organisation, supported local initiatives on GOC. In March 2022, twenty-five primary healthcare organisations received project funding to experiment with the translation and implementation of GOC in practice.

The objective of this study is to explore and understand the experiences of primary care organisations in the process of implementing GOC within their specific context.

## Method

### Design

This study followed a qualitative description research design to capture the experiences of project leaders in implementing GOC in their primary care context [17,18]. The aim was to provide a rich and detailed account of the experiences of the project leaders. Data were collected through field notes, a survey with open questions, and in-depth interviews to ensure data triangulation and analysed using an inductive thematic analysis [19].

The Standards for Reporting Qualitative Research (SRQR) checklist was used to ensure methodological rigour and to guide the reporting of this qualitative study [20].

### Research team

This research was carried out by an interprofessional research team, consisting of researchers with a background in general practice (3), occupational therapy (5) and sociology (2). All researchers have expertise in qualitative research. All researchers have done previous research in the context of primary care. Two researchers are combining a job as researchers and as a primary care professional.

### Context

The Dr Daniël De Coninck Fund, which is part of the King Baudouin Foundation a Belgian philantrophy organisation, invests in high quality primary care in Belgium. The Fund supported local initiatives on GOC in 2022. In 2021, there was an open call where primary care organisations could apply for project funding, ranging from 5.000 to 30.000 euros. Each organisation had to write a project proposal that was evaluated by an interprofessional jury. Eventually, 25 organisations were selected and received funding in March 2022. The funding needed to be used to set up actions to implement the concept of GOC into their own context. Each project had their own focus and was different in scope, aim and target group.

Each project had one or two project leaders, these project leaders were the people responsible for the project. The project leaders had to participate in a minimum of three peer learning activities with the other projects. The projects formed a learning collaborative for GOC in Belgium where they could share their experiences. The Belgian health system is based on the principles of equal access and freedom of choice [21]. Overall, the health system in Belgium has a high quality and accessibility, however there are points for improvement such as continuity and coordination of chronic care, preventive care and socioeconomic inequalities [22]. Responsibilities are shared between the federal government and three territorially based regions (Flanders, Wallonia and Brussels) as well as three language based communities (Dutch speaking, French and German). The organisation of primary care is a responsibility of the communities and therefore differs across Flanders, the Dutch speaking part, Wallonia, the French speaking part and the German speaking community. The payment of primary care providers, however, is a Federal responsibility [23]. In Flanders, primary care is organised in 60 primary care zones, which serve a population of 75.000 to 125.000 inhabitants and aim to support integrated and intersectoral collaboration.

### Participants

Through purposive sampling, and more specifically maximum variation sampling, all the project leaders of the above mentioned call were contacted and selected to participate in the study to ensure a broad range of perspectives. In maximum variation sampling, a wide range of cases is selected to get variation on dimensions of interest [19]. This guaranteed that broad insights and rich information were captured on the implementation of GOC [17].

All project leaders were affiliated with a primary care organisation. Their role in the primary care organisation was diverse eg.: coordinator, health care professional, care coordinator, responsible for projects….

Ethical approval was granted from the Ethical Committee of the University hospital of Antwerp under project ID 3542. Participants were informed about the collection of data and gave written consent to collect the surveys and oral consent to participate in the interviews and observation.

### Data collection

**Figure 1:**
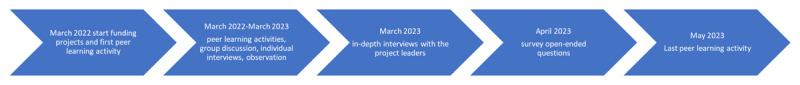
Timeline of key data collection moments.

A multi-method approach to data collection was used. Field notes, a survey with open-ended questions and in depth-interviews were collected to assure data triangulation [19]. Figure 1 provides an overview of the project timeline and key data collection moments.

Field notes were collected during observations and peer learning activities by LV, DB, RH, IH & IH. Field note were taken to describe the context and interaction among the participants as well as to document the researchers’ initial reflections. Field notes are a central component in qualitative research and origine from ethnographic research. Within qualitative research, they are used to provide a rich context for analysis [24]. An online survey, with open-ended questions, was distributed to the project leaders by email in January 2023, and collected by the end of April 2023. The survey was developed by LV, IH & PB (see Supplementary File 1). Between March and June 2023, semi-structured in-depth interviews were conducted by LV with Dutch speaking project leaders. These interviews were held online via Microsoft Teams, recorded with the participants consent and transcribed. An interview guide was developed and used (see Supplementary File 2). The interview began with the open ended question *How did the project go so far?* With follow-up questions exploring the participant’s responses in depth. Additional interview questions included: What lessons have you learned? What obstacles did you encounter and how did you address them? What has supported you? What advice would you give to others looking to implement GOC in their organisation? How can continuity and sustainability be ensured?

### Data analysis

Data analysis followed an inductive thematic analysis approach [25]. The analysis was conducted manually and facilitated by a data extraction table in Excel. The field notes, surveys and interview transcripts were read multiple times to ensure thorough familiarity with the data. To enhance the credibility and trustworthiness two researchers (LV&IH), trained in qualitative research, independently conducted the initial stages of analysis. Codes were generated from the data and organised into subthemes and overarching themes. The preliminary analysis and identified themes were discussed with the core research team (PB, JLB, IH, LV) and later with the broader research team (IH, DB, DVDV, RH, PDV). The themes were reviewed by the research team and the final themes were identified. To ensure trustworthiness and alignment with participants’ experiences, the themes were presented to the project leaders during the last peer learning activity in May 2023, serving as a member check. Table 1 gives an example of the data analysis process.

**Table 1:** Data analysis process.


DATA	CODE	SUBTHEME	THEME

I asked openly to them (the physicians) what they encounter in complex care and what they (the physicians) need. How could we respond to that with training?	Asking the questions ‘what do you need’ at the start of a training	Tailoring the training to the needs of the participants	Training people in GOC towards sustainable implementation


## Results

Table 2 provides an overview of the 25 projects and the types of data collected. Field notes were collected for all 25 projects. Twenty-two surveys were collected and twelve project leaders were interviewed.

**Table 2:** Overview primary care organisations, projects and data.


	PRIMARY CARE ORGANISATION	OVERVIEW PROJECT	AVAILABLE DATA F = FIELD NOTES S = SURVEY I = INTERVIEW

**1**	Patient organisation	A life path towards goal-oriented care on a human scale. To promote and explain the tools for more goal-oriented care using an example, in order to raise awareness among all stakeholders about their application. Organisation of a conference on GOC and workshops with a tool GOC for patients.	F, S, I

**2**	Peer support group	Welcome Home, a first step in peer support towards goal-oriented care. Use a tool to promote dialogue/collaboration between peers with mental vulnerability and care/welfare services, with a view to active co-decision making. Exploration with a tool GOC for citizens, together with volunteers.	F, S, I

**3**	Monodisciplinary healthcare centre	(Un)concerned pregnancy: integrating personal concerns into prenatal care. In addition to prenatal care/consultation, develop a tool to assess the needs/wishes of the pregnant woman/partner in advance, using a short questionnaire of concerns. Introduction of a GOC tool and peer learning activities for professionals in the centre.	F, S, I

**4**	Multidisciplinary healthcare centre	Healthcare Esperanto Methodology: a shared language for patients, caregivers and professionals. Exploring the methodology within the community health centre to contribute to multidisciplinary consultation and ownership of patients through a shared language around (complex) care questions. Training sessions for professionals and informal caregivers in the centre. Developing GOC processes.	F, S, I

**5**	Home care service	Goal-oriented care: care providers and the network as co-pilot for care recipients and informal caregivers. Motivating care recipients who are moving to a residential care centre/assisted living facility to express their concerns and encouraging care providers to respond flexibly to these concerns. Development and implementation of a training for professionals.	F, S, I

**6**	Home care service	‘Person-central’: implementing goal-oriented care in phases and processes Select and test methods with a group of clients/caregivers and provide training for employees to focus on the needs of clients/caregivers. Implementation of a tool GOC for patients and informal caregivers.	F, S, I

**7**	Flemish Primary care zone	Expedition goal-oriented care for primary care/welfare actors in Central West Flanders. With primary care actors, led by a guide, actively exploring the diverse landscape of concepts and tools regarding goal-oriented care for people with care/support needs. Coaching and training on GOC for professionals in a geographic region.	F, S, I

**8**	Flemish Primary care zone	Goal-oriented work in the Waasland: for a satisfied patient/client and care provider. Explain the principles of goal-oriented work/coaching professionals/central role of the patient to healthcare providers and encourage them to match care wishes and reality. Coaching and training on GOC for professionals in a geographic region.	F, S, I

**9**	Flemish Primary care zone	Primary care zone Scheldekracht is enthusiastic about goal-oriented care. From exploration to implementation. To ask the stakeholders involved about their views on (goal-oriented) care and presenting tools, and to support/discuss goal-oriented care in practice. Introduction on GOC for patients and professionals in a geographic region.	F, S, I

**10**	Training, scientific support and advocacy organisation (federation, professionnal associations…)	Goal-oriented care in general practice: further training/change of mentality of all employees. Develop a package on goal-oriented care in local quality groups to support general practitioners, based on their own practical experience, in the transition to goal-oriented care. Development of a training package GOC for general practitioners.	F, S, I

**11**	Health insurance company	Social work services introduce principles of goal-oriented care in care provision. Develop a training course to help health care workers put the care recipient at the centre within an interdisciplinary approach, aiming at quality of life of clients. Developing GOC processes and the organisation of a conference GOC for social workers.	F, S, I

**12**	Multidisciplinary healthcare centre	Better care follow-up of chronic patients in the community health centre. Thinking about developing shared care goals, to unblock seemingly inextricable situations and avoid difficulties. Developing GOC processes and tools.	F, S

**13**	Multidisciplinary healthcare centre	For holistic, accessible medicine where patient/life project is central. Empowering patients by informing them/treatment according to their priorities, and developing disease-specific procedures/tools tailored to each patient’s. Developing GOC processes and tools.	F, S

**14**	Multidisciplinary healthcare centre	Multidisciplinary working around patients’ life goals in community health centre. Collecting life goals from people with diabetes, people aged 70+ and people with chronic illness, coding them in the patient record, and guiding/supporting the team. Developing GOC processes and the implementation of a GOC tool in the centre.	F, S

**15**	Residential care centre	The purpose of life is central to residential care: a multidisciplinary primary care approach. Tailoring care to the hopes, expectations and end of life of WZC residents, contributing to meaning for staff members, and sharing the experience gained. Developing GOC processes and tools.	F, S

**16**	Senior citizen organisation	My life book: ‘Will hospital/care center respect my taste, choices, wishes?’ Using an instrument from the elderly association Énéo, provide administrative information and start a dialogue with family members/professionals to ensure that the choices of the elderly are respected. Developing a GOC tool together with the seniors.	F, S

**17**	Counselling organisation for older people	How to guide the elderly to a suitable place to live, with a view to their self-determination? Inform the elderly, family members and professionals at an early stage about housing options for the elderly, and visit these with peers (older volunteers) to enable them to make an informed choice. Developing GOC tools.	F, S

**18**	Service for people with addiction	To be or not to be: making life goals of addicted patients visible through theatre work. Using the processing of testimonies from/by patients about their addiction/withdrawal process in podcasts/clips/a play as an educational tool and life goal. Group activities to enhance person’s capabilities to define their own goals.	F, S

**19**	Housing first organisation	Housing First reconnection team for former homeless people with mental and addiction problems. In addition to psychomedical-social teams, working on people’s life goals, through individual follow-up and participatory community activities. Group activities to enhance person’s capabilities and to follow-up the goals.	F, S

**20**	Home care service	Testing a person-centred approach in a home care service in Brussels. Introduce home care nurses to the Montessori method of assistance/care adapted to the elderly and/or people with loss of autonomy, with a view to change. Training for professionals.	F, S

**21**	Training, scientific support and advocacy organisation	Users and caregivers: partners in care in a community health centre. Organizing an action research and co-training on goal-oriented care, for an individual and interdisciplinary professional practice focused on the patient’s life goals. Inventory of existing tools on GOC across organisations and supporting member-organisations.	F, S

**22**	Training, scientific support and advocacy organisation	Living and growing old in health. To keep doing what you love! To raise awareness among the elderly to regularly optimize the required capacities for their life purpose, in order to maintain their well-being/independence in daily life. Workshops on GOC for older people. Local sessions to support networking between local professionals and authorities.	F, S

**23**	Multidisciplinary healthcare centre	The other conversation: is goal-oriented care difficult for socially vulnerable goal-oriented groups? Despite resistance from some physicians, as a care coordinator, continue to strive for the implementation of goal-oriented care at all levels/domains of the primary care organisation. Training sessions for professionals in the centre. Developing GOC processes.	F, I

**24**	Social housing company	3 Zen Age: Spending the autumn of your life in peace, in a group home Develop an Everecity housing project to bring together seniors and surround them with benevolent neighbours and specific health and psychosocial care, provided by professionals. Research for development of a participatory co-housing project for older people.	F

**25**	Advocacy, knowledge and network organisation	Putting goal-oriented care into practice locally in public residential care facilities. Organise learning community and offer training and inspiring practices, to write a personal, goal-oriented care story with care recipients. Coaching and training on GOC for professionals in Flanders.	F


Field notes (F) = 25 Survey (S) = 22 Interviews (I) = 12

Seven themes could be elicited from the data. Overall the project leaders had positive experiences with the implementation of GOC into their local context and organisation. Each theme Is highlighted with quotes from the project leaders.

### Theme 1: Related concepts as a foundation for GOC

**Figure 2:**
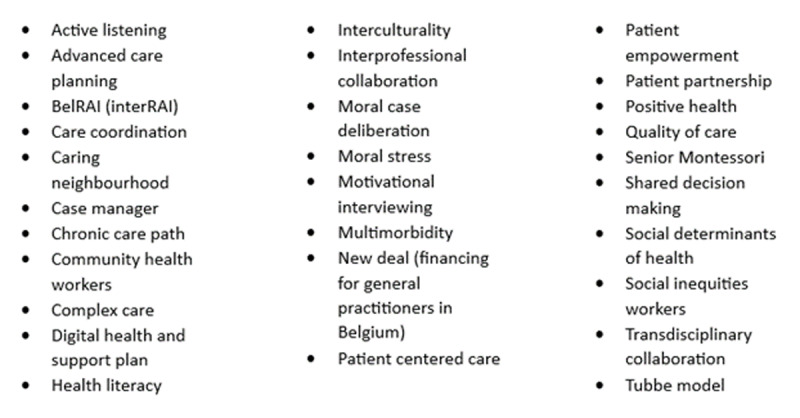
Concepts related to GOC (this list is non exhaustive).

Project leaders reported that starting with concepts familiar to professionals can be a useful strategy for implementing GOC. These related concepts serve as a foundation, helping to anchor the implementation process. Figure 2 illustrates examples of such concepts identified by project leaders as effective anchors for GOC. The implementation process begins by using existing resources and gradually introducing new steps within existing workflows which helps to engage professionals. Project leaders observed that many professionals are already practicing elements of GOC, either conscious or unconscious. The challenge was to avoid overwhelming the team with new procedures, as the perception of additional work could provoke resistance. By emphasising that GOC is not entirely new but rather an extension of current practices, resistance to change can be mitigated.

“Goal-oriented care is so closely aligned with the social worker’s DNA, there are so many basic elements that we recognise or already do. You don’t have to come very far to work together with those people because it is already the way things are. This means that the concept is well received.” (health insurance company; interview).

### Theme 2: Engaging in a dynamic implementation process

Project leaders perceived the implementation of GOC as an iterative and reflective process. The project leaders shared the need of going progressively and taking time to roll out various actions. They emphasised that by starting small and taking small steps, the project could grow. A lever in the implementation process was the project funding. Without funding, the project leaders won’t be able to invest time and people to implement GOC.

“Implementing the project at the team level requires a lot of patience, motivation, the need to establish fertile ground for change, to balance the relationships between professionals -each with a different background- and between the professional and the individual.” (multidisciplinary healthcare centre; survey)

During the implementation process, resistance was faced. One of the reasons formulated by the project leaders is that the term GOC is not appealing to everyone. Not every primary care professional feels connected to it due to the word ‘care’. Some professionals also perceive that GOC does not apply to them or believe that they are already practising it. Project leaders reported that resistance is not only perceived as an obstacle, but can also serve as a lever. They found that facing resistance led to reflection, not only for themselves but also for others involved in the project. This reflection on why they were implementing GOC was beneficial for the overall progress of the project and resistance was used as an opportunity for learning.

“I think that last year, we wanted to adjust the methodologies too quickly. I believe we pushed for changes too hastily, even where we encountered significant resistance, trying to force a shift when the timing was not right. On the other hand, I do not regret that the work was done because it initiated a process of reflection. Our group realised that we should not dismiss resistance as non-existent. We first need to better understand that resistance we now comprehend it.” (health insurance company; interview)

In the implementation process, project leaders were struggling with whether to adopt a bottom-up or top-down approach. They reported that both are necessary for successful implementation and to address resistance. Top-down needs to give the mandate and opportunity to develop actions bottom-up. Bottom-up, there needs to be commitment to perform these actions.

“If your organisation does not agree to implement it, you may be convinced of it and see the added value, but if you are not given the opportunity to get started with it, then it will not work.” (primary care zone; interview)

### Theme 3: The strength of collaboration

The project leaders stressed the importance of collaboration within and across organisations. Within the organisation, the designation of an ambassador or a dedicated working group showed to be useful for maintaining focus on the process. Internal communication about the implementation of GOC from the start was reported, even if it is a pilot project.

“The advantage was that three colleagues had already attended a session on goal-oriented care through the primary care zone, and I see them somewhat as ambassadors for goal-oriented care beyond myself. And those were a doctor, a nurse, and a social worker. This also helped in getting doctors, who were somewhat resistant, to make that mental shift.” (multidisciplinary healthcare centre; interview)

“We probably should have set up a project support committee to involve more people internally and ensure that there were not just two people carrying out the project and keeping it alive.” (homecare service; survey)

Across organisations, different partnerships were perceived as catalysts for the implementation and continuation of GOC, particularly when these partnerships are established within the same region. Some projects asked experts or coaches on GOC to support their project, some projects brought together an advisory committee with partners. Project leaders experienced that involving partners is a challenge and it takes time to establish a network.

“There was a lack of response from local authorities and doctors. (…) Sometimes months passed between the various contacts and the start of an information session. It is a slow process, but it is important to follow their timetable so that we can continue to build the project with them and plan for continuity.” (Training, scientific support and advocacy organisation; field notes)

All the project leaders expressed the importance of the peer learning activities, organised by the Fund Dr. Daniel De Coninck. Peer learning activities occurred between multiple project leaders. These activities were perceived as inspiring and encouraging moments to discuss the implementation process. The participants strongly advised anyone starting to engage in peer learning activities.

“Meeting the other project leaders, working in subgroups, comparing ideas, theoretical input and the emergence of common difficulties were also great facilitators of the progress of the project.” (multidisciplinary healthcare centre; field notes)

“Join a network if it already exists. If necessary, think about building such a network. In any case, do not work alone.” (Training, scientific support and advocacy organisation; survey)

### Theme 4: Engage patients in the implementation process

Patient engagement was present in the projects. The project leaders from patient organisations underlined the importance of patient engagement as a foundation for implementation, starting from an equal relationship between the patient and the provider. In overall, the project leaders reported to be convinced that the quality of implementation is better in projects involving patients.

“In goal-oriented care, an equal relationship is important, this needs to resonate in the implementation of GOC.” (patient organisation; interview)

Patients were involved on different levels. Patients, and their informal caregivers, were invited to participate in training and were sometimes even involved as co-teachers. This was experienced as positive because of the unique view the patients gave during case discussions. In other projects, patients organised group sessions for other patients, in which they testified. Other projects made videos and podcasts with patient testimonies which were used to raise awareness among professionals or encourage other patients. Even the organisation of a symposium on GOC in which patients, informal caregivers and professionals were all part of the speakers and the audience was suggested as a means to engage patients in the process of GOC. A different level in which patients were involved was in the project follow-up by validating the tools and processes, methods or workshops and by being active members in the follow-up meetings. In one organisation, the participation of patients in the GOC implementing project led to the creation of a permanent patient committee.

“On the other hand, satisfied patients have been a real help in promoting the tools, mainly thanks to word of mouth.” (counselling organisation for older people; field notes)

Project leaders mentioned that involving patients in the project also comes with specific challenges. Their availability and health situation need to be taken into account. Some of the patients who participated in the projects needed to disengage for health reasons.

### Theme 5: Build a GOC vision within your organisation

To implement GOC, project leaders invested in writing a vision and mission statement supported by the whole organisation. By doing so, GOC is formalised and included in the strategic objectives of an organisation. Project leaders of smaller organisations recommend writing a vision statement with the whole team. Project leaders of bigger organisations wrote the vision statement with a working group. Some project leaders contacted an experienced coach on GOC to support them with the writing of a vision statement.

The project leaders experienced that a vision statement about GOC is supportive to further implementation. If the vision is not supported by the organisation, it is experienced difficult for GOC to be sustainably embedded in the organisation.

“The theoretical framework and the vision is truly needed (…) but the vision around goal oriented care at the organisational level is truly a prerequisite. I think, both the discussion around this and a framework and someone facilitating. I think this is important for us, in our organisation.” (multidisciplinary healthcare centre; interview)

Project leaders expressed that by actively engaging with GOC in practice, the values that are put forward in the vision of the organisation becomes more tangible and concrete. By doing so, the GOC project helped them to confirm the values and vision of the organisation.

“At an institutional level, our experience and learning have enabled us to put the association’s missions back into perspective. In particular, by giving concrete attention to the values advocated by the association through the use of tools.” (counselling organisation for older people; survey)

Not only a vision within an organisation, but also a shared vision between organisational partners was perceived as supporting. According to the project leaders, it is up to organisations to write their own vision but it can be inspiring to work together with other organisations and inspire each other.

### Theme 6: Using and adapting existing GOC tools and processes

In each project, tools for defining patient goals in practice were developed or existing tools and processes were adapted to better suit their organisational needs and context. For example life goals were added in patient health records, intake forms were redesigned to make goals visible, templates for multidisciplinary consultation were redesigned with a focus on goals.

“Therefore, we are going to screen our current procedures and where we can insert life goals and we are going to put them above the care goals.” (health insurance company; survey)

Merely developing tools and/or adjusting processes is not enough. Embedding these tools and/or processes within the organisation is also necessary. Project leaders emphasised the importance of having tools and processes that align with the culture and structure of an organisation, this allowed organisations to preserve their identity. Tools can contribute to the vision of GOC in practice, but they can’t stand alone. The project leaders reported that training is needed for professionals who use a tool.

“Don’t believe in a miracle tool. The key is adaptation.” (home care service; interview)

### Theme 7: Train people in GOC towards sustainable implementation

Training for primary care professionals, patients and informal caregivers was commonly used as a strategy for the implementation of GOC. Training was mostly offered within a single organisation, or across organisations within the same region.

Project leaders discussed the importance of tailoring the content of the training to the needs of the participants. The project leaders highlighted that a theoretical base is necessary, but enough time is needed to focus on practical applications. In this context, case discussions and peer learning activities are perceived as useful and appealing to the participants. During training, the attitude of putting the patient central should be the primary focus.

“If you want it to be implemented, it has to match the needs of the practice. So truly take the time to have a conversation with your target group, where are your needs and then carefully put forward, could this formation of goal oriented care be supportive, which elements are or are not. Instead of saying here is what we’re going to work with and we will roll it out.” (Training, scientific support and advocacy organisation; interview)

Project leaders reported struggling to motivate professionals for training. They reported that if professionals clearly see the added value of the training and what’s in it for them, their motivation to participate will be higher. Barriers why professionals don’t participate may be content related, such as not enough interdisciplinary case discussions but also practical, such as accreditation, location and time.

“It is not easy to make someone else’s personal benefit clear or felt. That is one thing for me, how to do that, I have not figured out yet.” (monodisciplinary healthcare centre; interview)

When providing training within an organisation, the project leaders reported the added value of training the whole organisation, and not only focused on healthcare professionals. By including administrative staff, cleaning staff, receptionists, volunteers and supervisors the cultural change within the organisation is further supported.

## Discussion

This study identified seven themes that supported the implementation of GOC in primary care settings. The results highlighted that starting from related familiar concepts can serve as a foundational anchor for GOC implementation. This approach allows for the introduction of GOC within existing frameworks reducing the perceived burden of new procedures and mitigating resistance. The implementation process was found to be cyclic and reflective, where resistance, rather than just being an obstacle, serves as an opportunity for improvement. This aligns with literature describing GOC as a paradigm shift that inherently involves resistance [26].

Collaboration emerged as a facilitator in the implementation process, particularly the involvement of patients as active partners. Ambassadors—individuals who are already practicing GOC—were identified as key motivators within teams. This finding is consistent with other studies, that emphasizes the role of opinion leaders and champions in facilitating implementation [27,28,29]. Opinion leaders are identified as change agents that could remove barriers to change. We believe that the ambassadors, identified in our results, acts as a liaison between project leaders and primary care professionals in practice. Furthermore, the engagement of patients in the implementation process was especially valued for the unique perspectives they bring, underscoring the importance of their role as consultants, collaborators, and partners. Although patient engagement is increasingly recognized in health research [30,31], there remains a need for a validated framework to sustainably embed patient engagement in health research [32].

Project leaders stressed the importance of creating a vision and organising training within the organisation and adapt tools to their specific context. It highlights the importance of the both sides of integrated care, as modelled in the Rainbow Model of Integrated Care (RMIC). The RMIC provides a useful framework for understanding the dual focus required in implementing GOC: functional and normative integration [9]. Functional integration focusses on supportive functions to facilitate the process of care, it involves adapting tools and processes to support GOC. Normative integration includes the shared concept, it focusses on developing a shared vision and common frame of reference within the organisation. Both functional and normative integration are necessary to achieve a successful implementation and integrated care. Their interaction is key [9].

When looking at implementation actions on GOC, it’s experienced that a vision about GOC is supportive for a sustainable implementation. This is an action on normative integration. To implement this vision, tools and processes on GOC are developed and/or adapted by the projects. This is seen as functional integration. By providing tools for professionals, GOC is facilitated in the process of care. However, as also stated in the results, the use of tools alone is not sufficient for implementation. Tools could support implementation if they become normalised practice within an organisation. Training is necessary to learn how to use these tools and the attitude of GOC, training is seen as normative integration. The provision of training, which integrates both theoretical and practical aspects, was seen as essential in normalising GOC practices within organisations. This approach aligns with the concept of organisational health literacy, which advocates for organisation-wide efforts to facilitate cultural change and support patient care [33].

The study also links to the findings of Huybrechts et al., who identified commitment, recognition, and coordination as the three primary drivers of GOC implementation at the macro level [11]. In our research, these drivers were also evident at the micro and meso levels. Commitment was demonstrated by project leaders who initiated GOC projects with intrinsic motivation, further bolstered by the allocation of financial resources. Recognition of GOC varied, with project leaders acknowledging its value, but some professionals resisted due to a lack of recognition. One way to address this resistance was by linking GOC to already established concepts. Coordination was facilitated at multiple levels, including macro-level support from the Dr. Daniël De Coninck Fund and peer learning communities among project leaders, which fostered collaboration and shared learning.

### Recommendations for practices, policy and research

Based on the results and the discussion, six recommendations are proposed to support the future implementation of GOC.

1. Identify and use related, familiar concepts as anchors for GOC implementation to build on existing knowledge and practices.
2. Engage in a dynamic process: begin with small steps and incorporate reflection throughout the implementation process to adapt and improve.
3. Collaborate with partners: involve relevant partners within and across organisations and create or participate to local peer-learning activities.
4. Engage patients: actively engage patients in the GOC projects, recognising their potential roles as collaborators.
5. Focus on functional and normative integration: develop a shared vision and adapt tools and processes to embed GOC within the organisation. Training should address both functional and normative aspects to ensure comprehensive implementation.
6. Invest in project funding: continue providing project funding to support primary care organisations in dedicating time and resources to sustain GOC implementation.

### Strengths and limitations

The strength of the study is in its representation of a variety of projects within primary healthcare providing a rich source of information on the implementation of GOC. Data-triangulation and the involvement of a multidisciplinary research team contributed to the credibility and trustworthiness of the findings. This is among the first studies to explore organisational implementation of GOC, rather than focusing solely on individual professionals’ practices with patients.

However, the study has limitations. The project leaders were followed only for 14 months (March 2022–May 2023), which is a relative short period of time for assessing the sustainability of GOC implementation as such process generally takes multiple years [34]. Interviews were only collected from Dutch speaking participants, because French is not the mother tongue of the researcher conducting the interviews. This could induce bias, because no French interview data was collected and analysed. The selection of participants may have introduced selection bias, as they volunteered to implement GOC. According to Rogers’ Diffusion of Innovation theory, individuals vary in their openness to implement an innovation, with the so called ‘innovators’ and ‘early adopters’ more open to innovation [35]. In this study, the participants are considered as ‘early adopters’ because they volunteered to implement GOC. Primary care organisations that are less open to implementation might yield different results and recommendations.

Additionally, this study did not aim to build a theoretical framework for implementation research. Analysing the data deductively using established frameworks such as Rogers diffusion of innovation [35] or, the Promoting Action on Research Implementation in Health Services (PARIHS) framework could lead to further insights into GOC and primary care innovations [36,37]. Other recommendations for further research could focus on how patients perceive the implementation of GOC and the skills patients need to address their goals. It would also be interesting to focus on the role of higher education and how to train future healthcare professionals to implement GOC [38].

## Conclusion

The 25 primary care organisations each implemented GOC in unique ways within their specific context. Seven recommendations were formulated that could support the implementation of future projects. The overall drivers for GOC implementation – commitment, recognition and coordination – were consistent with those identified in existing literature. Successful implementation requires more than developing and adapting tools; it necessitates translating theory into practice, creating a shared vision and engaging in both functional and normative integration.

The focus in implementation is not merely on developing and adapting tools but also on translating theory into practice and creating a common GOC vision. Engaging in both functional and normative integration is key for successful implementation.

## Additional Files

The additional files for this article can be found as follows:

10.5334/ijic.8983.s1 Supplementary File 1. Survey.

10.5334/ijic.8983.s2 Supplementary File 2. Interview Guide.
