# Supplementary material for: Implementation of Goal-Oriented Care in Belgium: Experiences From 25 Primary Care Organisations
Source: Int J Integr Care. 2025 May 2;25(2):7. doi: 10.5334/ijic.8983 (PMC12050686; doi:10.5334/ijic.8983)
Supplement: Supplementary File 1. — Survey. [file ijic-25-2-8983-s1.pdf]

Supplementary File 1: survey

General project description

**Name of the project**
**Project leaders and contact details**
**Challenge: what would you like to achieve within 1-2 years from the start of the project?**
**Problem definition**<br><br>**What problems underlie this initiative? And why did you choose this approach? What events influenced the choice to engage in this project?**
**Target group(s) and their needs**<br><br>**Who does your initiative address as a priority? How can goal oriented care better meet the needs of these target group(s)?**
**Context in which the project started**<br><br>**Describe below which context elements are important**

**The partners in the project and their roles**

What can we learn?

**The proces**<br><br>**How did your initiative go?**
**What phases did you go through? Were these originally planned or did you make changes as you went along? Describe the concrete outcome of each phase.**

In your efforts to implement goal-oriented care...

**What surprises or unwanted effects did you encounter? (both positive and negative)**

**What resources and competences can you rely on to move your initiative forward? What capabilities support project progress?**
**What obstacles did you encounter?**
**Did you manage to overcome these obstacles? If yes, which ones and how did you do it? If no, which ones didn’t and why not?**
**What are you incredibly proud of?**
**What did you learn about goal-oriented during project implementation (at the organizational, team, professional, individual level)?**

**Short-term effects**<br><br>**What effects do you see among the target group(s) reached?**<br>**What effects do you see among the project partners?**<br>**What effects do you see among colleagues?**
**In what ways did you involve individuals with care and support needs and their personal networks?**

Future

**How will you ensure the continuity of your initiative in the future? What are the necessary conditions to make goal-oriented care sustainable in your organization/network ?**
**What are the internal options for ensuring continuity?**
**What ideas do you have to ensure continuity if there are no internal opportunities?**

**What tips & tricks would you like to share with an individual caregiver who wants to get started with goal-oriented care? What should you pay attention to?**
**What tips & tricks would you like to share with an organization who wants to get started with goal-oriented care? What should you pay attention to?**
**What encouraging messages would you like to give to a person who wants to get started with goal-oriented care? How to motivate or empower this person to convince them that it is worth investing in goal-oriented care.**
