# Supplementary material for: Implementation of Goal-Oriented Care in Belgium: Experiences From 25 Primary Care Organisations
Source: Int J Integr Care. 2025 May 2;25(2):7. doi: 10.5334/ijic.8983 (PMC12050686; doi:10.5334/ijic.8983)
Supplement: Supplementary File 2. — Interview Guide. [file ijic-25-2-8983-s2.pdf]

Supplementary File 2: interview guide

How did the project go so far?

Which lessons have you learned during the project?

How did you deal with obstacles?

What supported you?

Do you see some effects? Can you give an example?

Which tips would you give to others who want to implement goal-oriented care within their organization?

How will you guarantee the continuity or sustainability of the project?

Are there some more things you want to discuss or questions you have?
